# Topography-specific spindle frequency changes in Obstructive Sleep Apnea

**DOI:** 10.1186/1471-2202-13-89

**Published:** 2012-07-31

**Authors:** Suzana V, Diego Z Carvalho, Emerson L de Santa-Helena, Ney Lemke, Günther J L Gerhardt

**Affiliations:** 1 Sleep Laboratory, Division of Pulmonary Medicine, Hospital de Clínicas de Porto Alegre, Rua Ramiro Barcelos 2350/sala 2050, Porto Alegre, RS, 90035-003, Brazil; 2 Department of Physics, Universidade Federal de Sergipe, Säo Cristóvao, Brazil; 3 Department of Physics and Biophysics, Institute of Biosciences, Univ Estadual Paulista (UNESP), Botucatu, Brazil; 4 Department of Physics and Chemistry, Universidade de Caxias do Sul, Caxias do Sul, 95001-970, Brazil

**Keywords:** Time series, Matching pursuit, EEG, Sleep spindles, OSA

## Abstract

**Background:**

Sleep spindles, as detected on scalp electroencephalography (EEG), are considered to be markers of thalamo-cortical network integrity. Since obstructive sleep apnea (OSA) is a known cause of brain dysfunction, the aim of this study was to investigate sleep spindle frequency distribution in OSA. Seven non-OSA subjects and 21 patients with OSA (11 mild and 10 moderate) were studied. A matching pursuit procedure was used for automatic detection of fast (≥13*Hz*) and slow (<13*Hz*) spindles obtained from 30min samples of NREM sleep stage 2 taken from initial, middle and final night thirds (sections I, II and III) of frontal, central and parietal scalp regions.

**Results:**

Compared to non-OSA subjects, Moderate OSA patients had higher central and parietal slow spindle percentage (SSP) in all night sections studied, and higher frontal SSP in sections II and III. As the night progressed, there was a reduction in central and parietal SSP, while frontal SSP remained high. Frontal slow spindle percentage in night section III predicted OSA with good accuracy, with OSA likelihood increased by 12.1*%*for every SSP unit increase (OR 1.121, 95% CI 1.013 - 1.239, p=0.027).

**Conclusions:**

These results are consistent with diffuse, predominantly frontal thalamo-cortical dysfunction during sleep in OSA, as more posterior brain regions appear to maintain some physiological spindle frequency modulation across the night. Displaying changes in an opposite direction to what is expected from the aging process itself, spindle frequency appears to be informative in OSA even with small sample sizes, and to represent a sensitive electrophysiological marker of brain dysfunction in OSA.

## Background

Obstructive Sleep Apnea (OSA) is a pathological condition characterized by repetitive episodes of complete or partial upper airway obstruction occurring during sleep, often resulting in reductions in blood oxygen saturation and usually terminated by brief arousals [1]. Sleep becomes lighter, more fragmented and less efficient. Consequences are numerous and include sleepiness, impaired memory, depression, decreased quality of life and increased accident and cardiovascular risk.

OSA is most incident around the transition from middle-age to old age [1]. This is a life period when changes in non-REM (NREM) sleep patterns which are traditionally associated with OSA may be expected from the aging process itself [2,3]. Older age groups also show wider variance in NREM sleep architecture variables [2,3]. Usefulness of conventional sleep parameters in OSA investigation may thus be limited, or large study sample sizes may be required.

The best studied sleep microstructure element is the NREM sleep spindle. Spindles are believed to be critically implicated in sleep maintenance, memory consolidation and learning processes [4,5]. Spindle oscillatory frequency increases with age [6,7] and decreases in OSA [8]. These changes in opposite directions suggest that spindle frequency may be particularly informative in the context of OSA. Spindles undergo homeostatic and circadian regulation. In healthy controls, spindle frequency is increased towards the end of the night, when homeostatic sleep pressure is expected to be at its lowest [9-11]. Subjects with OSA, however, apparently maintain low spindle frequency throughout the night [8]. OSA-associated spindle abnormalities are therefore suggestive of structural changes in spindle-generating neuronal circuits and/or impairment of regulatory homeostatic mechanisms [8,12].

Studies of sleep spindles in OSA have been limited to information obtained with spindle frequency being assumed as an unimodal variable, and without consideration of the important influence of scalp topography on spindle frequency distribution [8,12]. Spindles are believed to have distinct, topography-dependent oscillatory regimens. Slow spindles (around 12Hz) prevail on anterior scalp positions. Fast spindles (around 14Hz) are more prominent on parietal locations. Central, classical sleep scoring channels display a mixture of these two spindle types [13-16]. Slow and fast spindles differently undergo modulatory changes in the course of sleep [9,17] and appear to have distinct functional properties [18].

In this study, sleep spindle frequency distribution is investigated in patients with mild and moderate OSA, considering scalp topography and changes across the night.

## Methods

### Subjects and recordings

Cases were prospectively and consecutively enrolled from the series of patients with suspected OSA [1] who underwent polysomnography (PSG) investigation in a university hospital-based sleep clinic (HCPA) between April 2007 and July 2009. All subjects provided informed written consent and the study was approved by the local ethics committee. Inclusion criteria were age between 34*y* and 60*y*, no previous treatment for OSA and no alcohol or substance abuse. A total of 45 patients were initially enrolled on the study. Subsequent to the PSG examination, 24 patients were excluded from analysis due to abnormal EEG activity (1), technical artifact (1), insufficient sleep (2), current benzodiazepine intake (5) and global apnea-hypopnea severity index (AHI) ≥30 (15). On the basis of global AHI index [1], the remaining 21 study subjects were categorized as non-OSA (No) (AHI<5), 7 subjects; mild OSA (Mild) (AHI 5−14), 11 subjects; and moderate OSA (Mod) (AHI 15−29), 10 subjects.

On the study day, subjects were requested to refrain from naps, exercises, alcohol and caffeinated drinks. Upon arrival at the sleep laboratory, neck circumference, height and weight were measured. Subjects were then requested to complete routine questionnaires addressing sleep habits, medication regimen and medical problems, including history of neurological disease. Subjective sleepiness was assessed with the Epworth Sleepiness Scale (ESS) [19], Brazilian version [20].

Continuous recordings were performed during the usual sleep period (23:00-07:00 h) on a 16 bit resolution digital system (Deltamed, Racia-Alvar, France). The recording protocol followed standard guidelines [21] including information on scalp EEG, eye movement, chin and leg electromyogram, electrocardiogram, snoring, airflow by oronasal thermistor, thoracic and abdominal respiratory effort, body position and pulse oximetry. Silver electrodes were placed over 10 standard 10-20 IS EEG positions (F3, C3, P3, O1, A1, F4, C4, P4, O2, A2). Initial impedances were below 10Kohms. The signal was acquired with 256Hz sampling rate, filtered at 0.5-35Hz and analyzed off-line using Coherence 3NT software version 4.4 (Deltamed, France). Sleep stages, arousals and respiratory events were visually scored by a trained polysomnographer in accordance with standard recommendations, applying obstructive hypopnea rule 4B [21].

### EEG sample

Each subject contributed with 30min of non-REM sleep stage 2 (N2) from initial (I), middle (II) and final (III) recording sections (10min from each section). Study epochs were sequential, but not necessarily consecutive, as 30s epochs containing excessive technical artifacts or any arousals, apnea or hypopnea events were excluded from analysis. Since faster alpha activity (typical of waking state) and lower sigma activity (typical of slow spindles) lie in the same (11-13Hz) frequency range, and since respiratory events have been shown to affect EEG frequency even in the absence of visually detected arousals [22], this measure, which excluded severe OSA subjects from the study, had the purpose of minimizing the potential confounding effect of alpha activity over the automatic detection of slow spindles. Signal analysis was performed on left and right frontal (F3, F4), central (C3, C4) and parietal (P3, P4) EEG channels referenced to (A1+A2)/2.

### Automatic spindle detection

**Figure 1:**
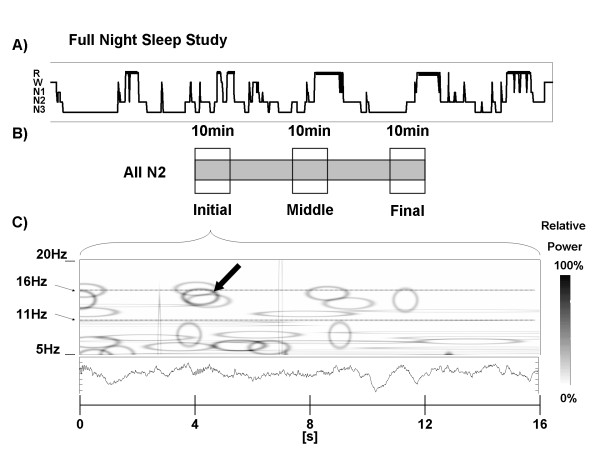
**Procedure employed for the automatic spindle detection. A**) Hypnogram representing one full-night recording (EEG time series), which was segmented into 2048 digital point-juxtaposed bins and subjected to matching pursuit signal decomposition. Atoms fulfilling study criteria (hereafter called spindles) filtered and collected in the procedure. **B**) Thirty minutes of N2 sleep fulfilling study criteria selected from initial, middle and final sleep study sections (10min each). **C**) Detail showing 16s of the original time series with the corresponding time-frequency representation in the Wigner plane. Each MP atom was represented as a hollow ellipse corresponding to its (time, frequency) HW and its relative amplitude (voltage) was indicated by color intensity. Only one atom in this figure (darkest ellipse marked with arrow) fulfilled all selection criteria and was considered as a valid sleep spindle. N2, NREM sleep stage 2; MP, Matching Pursuit; HW, half-width (see text).

Signal analysis was carried out with a matching pursuit (MP) program obtained from http://eeg.pl[23]. MP has been previously described in detail [24,25] and shown to be suitable for sleep spindle representation [23,26-28]. MP is not a transform, it is an adaptive approximation procedure, whereby the original signal is decomposed into waveforms corresponding to a set of fundamental functions belonging to a large dictionary. In the case of this particular algorithm, the dictionary corresponds to a large set of Gabor atoms, which are plane waves modulated by a Gaussian function. The original signal can thus be represented as a set of atoms in a time-frequency plane (Wigner plane, see Figure 1) where atom amplitude is related to signal energy (voltage). If a signal structure does not correlate well with any particular function, decomposition will result into a number of non-relevant elements and information will be diluted. After subsampling to 128Hz, each whole-night EEG series was segmented into juxtaposed bins of 2048 digital points and subjected to MP decomposition with a dictionary size of 1^05^ atoms, stopping at 96 iterations. Each atom obtained with MP has a central point in time and frequency, and time and frequency half-widths (HW) corresponding to ±*σ*on a gaussian curve. Duration HW can be used as one parameter for atom selection. Atoms with duration HW between 0.5s and 2s and central frequency between 11Hz and 16Hz, hereafter called spindles, were collected in the procedure. It should be emphasized that an individual MP atom fulfilling detection criteria is not conceptually equivalent to a visual sleep spindle, and the procedure is robust and reliable at the statistical level. Spindles were further divided into slow (<13*Hz*) and fast (≥13*Hz*) types according to central frequency.

MP performance has been previously shown to strongly depend on the choice of voltage threshold for sleep spindle detection [29]. Sensitivity decreases and specificity rises as voltage threshold is increased [28]. In order to ensure high specificity, analyses were performed for the top 20*%*amplitude spindles. This threshold was chosen after systematic testing of MP performance (with the detection parameters used here) on another sleep EEG sample (training dataset) pertaining to 9 healthy young subjects, where 513 sleep spindles had been visually identified during NREM sleep stage 2 [28]. After Receiver-Operator Characteristics (ROC) curves were built according to voltage threshold variation (see Additional file 1: Figure S1), a 20*%* amplitude threshold was verified to correspond to 96*%*MP specificity on the training dataset. An additional test of the false rate of spindle detection on the present data was carried out on 10-min N2 samples obtained from 3 subjects, one from each study category. From this sample, a polysomnographer blindly selected every spindle. False spindle detection by MP was respectively 11.1*%*, 9.7*%* and 12.5*%* for a non-OSA, a mild and a moderate OSA subject. Voltage threshold was also individualized in order to account for inter-subject spindle amplitude variability [30-32].

The problem of MP analysis can be classified as a bag of tasks [33], since it is performed through a parallel, independent set of tasks with high computational cost, whereas computational requirements for performing integrative analysis of results are negligible. Task scheduler Condor [34] was employed in the coordination of the time series analysis submission. Infrastructure details may be obtained in (Iope et al, 2010) [35]. Computational analysis was performed at São Paulo State University (UNESP) Center for Scientific Computing (NCC/GridUNESP).

### Statistical analysis

Non-parametric methods were used for group comparison of demographic and sleep architecture data, due to the limited number of subjects and asymmetrical (non-Gaussian) distribution of those variables. Gender proportions (male/female ratio) in the three studied groups were compared by means of Chi-square tests, while other group demographic and sleep characteristics were tested with the Kruskal-Wallis analysis of variance followed by Dunn’s post-hoc pairwise comparisons.

In a preliminary step, spindle number, duration, voltage and frequency distributions were obtained for single EEG channels. Spindle density was defined as the averaged spindle number per minute/channel, and compared among groups with ANOVA followed by Tukey’s post-hoc test. All spindle characteristics were then compared between homotopic brain locations across hemispheres. All variables with the exception of voltage were statistically similar between hemispheres. Voltage threshold was therefore individualized for every channel, and atoms representing sleep spindles were pooled together for frontal (F3∪F4), central (C3∪C4) and parietal (P3∪P4) regions. This measure provided a reliable description for sleep spindle behavior over the afore-mentioned sites, avoiding the excessive decrease in statistical power that might have resulted from family-wise error rate control by Bonferroni correction applied to a higher number of comparisons. After duration, voltage and frequency distributions were verified to be non-normal (D’Agostino & Pearson omnibus normality test), they were tested with the Kruskal-Wallis analysis of variance followed by Dunn’s post-hoc pairwise comparisons. Unless otherwise specified, these results are expressed as median (interquartile range, IQR) on text.

While sleep spindle duration and voltage correspond to left-skewed unimodal curves [36], frequency distribution is visually non-unimodal. This behavior was analyzed with the Dip test of unimodality [37]. In the dip statistic, the maximum difference between an empirical distribution and a unimodal distribution that best fits that empirical distribution is calculated for n observations (sample size). A uniform distribution is considered as the null hypothesis. Dip values approaching zero carry the highest likelihood of unimodality, and the p-value indicates the probability of non-unimodal distribution. Results of the Dip test are shown in Table 1. Frequency distributions were, in the large majority (78*%*), non-unimodal at the 0.9 probability level, indicating that variations in frequency medians are only partially informative and result from variability in the proportions of at least two (fast and slow) spindle populations. As a result, group comparisons were carried out considering slow and fast spindle percentages.

**Table 1:** Dip test of unimodality in spindle frequency distribution across AHI groups, scalp locations and night sections


		**Non-OSA (7)**		**Mild OSA (11)**		**Mod OSA (10)**	
		**Dip value**	**P-value**	**Dip value**	**P-value**	**Dip value**	**P-value**
		**(sample size)**		**(sample size)**		**(sample size)**	
Location	Night Section						
Frontal	I	0.03 (334)	0.81	0.03 (465)	**0.97**	0.03 (423)	**0.91**
	II	0.04 (250)	**0.98**	0.03 (439)	**0.99**	0.03 (390)	**0.90**
	III	0.03 (324)	**0.91**	0.03 (446)	**0.99**	0.03 (279)	0.88
Central	I	0.03 (326)	0.86	0.03 (426)	**0.94**	0.03 (472)	**0.98**
	II	0.03 (296)	**0.92**	0.02 (456)	0.73	0.04 (472)	**>***0.99*
	III	0.02 (366)	0.79	0.03 (486)	**0.99**	0.04 (351)	**>***0.99*
Parietal	I	0.03 (383)	**0.94**	0.03 (386)	**0.96**	0.03 (413)	**0.96**
	II	0.03 (438)	**0.99**	0.02 (460)	**0.93**	0.03 (415)	**0.99**
	III	0.03 (377)	**0.91**	0.02 (489)	0.65	0.06 (327)	**>***0.99*

AHI, Apnea-Hypopnea Index; Mod, moderate OSA; sample size, number of sleep spindles under study; Location, scalp EEG topography; total, (I+II+III) sample; I, II and III, initial, middle and final night sections, respectively. Bold indicates non-unimodal distributions at the 0.9 probability level. Frequency distributions were non-unimodal in the large majority (78*%*) of comparisons.

Slow spindle percentage (SSP) was compared among and within groups for topography and night section by means of Chi-square tests with Bonferroni correction for multiple comparisons. Statistical significance was assumed for two-tailed p-values <0.05. In order to identify whether SSP predicted OSA, a binary logistic regression analysis was performed for every topography and night section, applying the Enter method, with a 0.5 classification cut-off point and 20 maximum iterations. If predictive, a ROC analysis was performed in order to assess its diagnostic value. The dependent variable was OSA (AHI≥5). Due to the low sample size, the independent variables were limited to SSP and BMI. Analyses were performed with Mathematica (Wolfram Research Inc., Champaign, IL, USA), R (http://www.R-project.org) and SPSS V.17 for Windows (SPSS Inc., Chicago, IL, USA) statistical packages.

## Results

Demographic and sleep characteristics of study participants are shown in Table 2. There were no significant inter-group differences in age, gender, BMI, sleepiness (ESS), sleep architecture, mean or minimum NREM O2% saturation. Arousal index was higher in moderate OSA when compared to non-OSA subjects. Concerning medication use, non-OSA subjects were under allopurinol (1), angiotensin converting enzyme inhibitors (3), betablockers (3), thiazide diuretics (3), nonsteroidal antiinflammatory drugs (1), omeprazol (1), statins (1), tricyclic agents (TCAs) (1) and warfarin (1). Subjects in the mild OSA group were taking alendronate (1), allopurinol (1), angiotensin converting enzyme inhibitors (3), betablockers (1), beta2-selective agonists (3), calcium-channel blockers (2), thiazide diuretics (3), ipratropium bromide (1), omeprazol (1), statins (1), TCAs (2) and warfarin (1). Subjects in the moderate OSA group were making use of alendronate (1), allopurinol (1), antiretroviral agents (1), beta2-selective agonists (3), omeprazol (2) and TCAs (2).

**Table 2:** Demographic and sleep characteristics across groups


	**Non-OSA**	**Mild OSA**	**Mod OSA**	**P-value**	**Group differences**
Sample size	7	11	10		
Age, y	46.0 (5.7)	51.1 (6.8)	52.2 (8.6)	0.25	ns
Male, %	4 (57.1)	4 (36.4)	8 (80.0)	0.15	ns
BMI,(*kg*/^*m*2^)	30.5 (5.6)	30.0 (3.2)	28.4 (3.2)	0.54	ns
ESS, units	12.0 (4.3)	9.3 (6.4)	11.5 (4.2)	0.58	ns
Polysomnography Data					
TST, min	404.0 (61.6)	401.3 (44.0)	395.7 (47.9)	0.73	ns
Sleep efficiency %	85.1 (13.3)	88.0 (6.2)	84.7 (11.3)	0.85	ns
N1 sleep %	15.1 (8.8)	17.1 (8.8)	18.6 (6.8)	0.46	ns
N2 sleep %	44.5 (12.6)	37.5 (13.2)	41.5 (7.5)	0.56	ns
N3 sleep %	26.5 (12.3)	32.3 (13.7)	26.5 (8.2)	0.60	ns
R sleep %	13.9 (5.3)	13.2 (5.3)	13.4 (4.1)	0.98	ns
Arousals, events/h	18.7 (7.9)	22.8 (5.8)	30.6 (8.7)	0.02	Mod > Non-OSA
AHI	2.8 (1.4)	9.4 (3.0)	18.3 (3.6)	<0.0001	Mod > Mild
Mean NREM O2% sat	94.4 (1.8)	93.7 (2.1)	94.6 (1.0)	0.48	ns
Minimum sleep O2% sat	89 (12.0)	83.5 (6.75)	85 (8.0)	0.233	ns

Data presented as mean (standard deviation) unless otherwise specified. Mod, moderate OSA; BMI, body mass index; ESS, Epworth Sleepiness Score; TST, total sleep time; Sleep efficiency, percentage of TST per total recording time; N1-N3 sleep, NREM sleep stages; R sleep, REM sleep stage; sat, saturation; AHI, Apnea-Hypopnea Index; ns, non-significant; Mod, moderate OSA.

### General spindle characteristics

Considering all night sections, central channel spindle density was similar among groups (2.35 (0.69)/min for non-OSA, 2.07 (0.81)/min for mild, and 2.13 (0.91)/min for moderate OSA) (*F*=0.525; *df*2; *p*=0.595). Median spindle duration was also similar among groups (0.95 (0.54)*s*, 0.94 (0.59)*s*, and 0.91 (0.59)*s*, respectively); (*K*−*W*=0.387; *df*2;*p*=0.824). Median voltage was 44.64 (19.15)*μV* for non-OSA, similar in mild 43.69 (27.28)*μV*, and lower in moderate OSA (39.30 (12.56)*μV*); (*K*−*W*=77.014; *df*2; *p*<0.001).

### Non-unimodality in spindle frequency distribution

**Figure 2:**
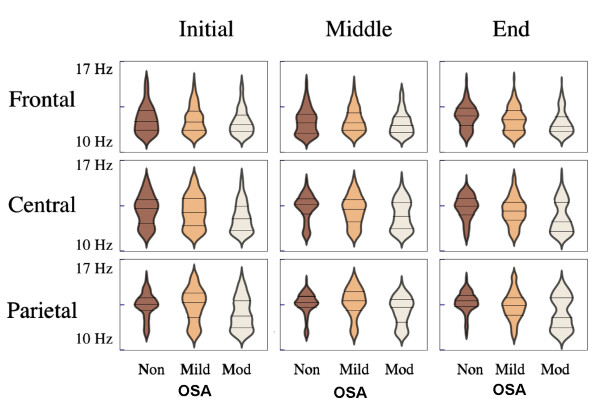
**Sleep spindle frequency distributions represented through violin plots [**[38]**], with shape width expressing spindle count grouped within 0.12Hz juxtaposed class intervals according to central frequency.** Distributions were largely non-unimodal. Compared to non-OSA subjects, in the beginning of the night, Moderate OSA patients showed larger contributions of slow spindles in central and parietal regions. As the night progressed, Moderate OSA patients showed spindle frequency changes that were topography-specific, with a relative reduction in the proportion of slow spindles in central and parietal regions (especially in the intermediate night section) whereas in frontal regions, sleep spindles remained slow. Horizontal marks indicate median and interquartile ranges. Non, non-OSA; Mod, moderate OSA.

Figure 2 shows spindle frequency distribution in OSA and non-OSA subjects, in different scalp locations and night sections. Group frequency medians, which were in the range between 12.01 and 13.82Hz, largely corresponded to different combinations of a slow (11.0-11.5Hz) and a fast (12.8-14.5hz) modal peak. In order to enable comparisons with other studies, median frequency results are available in Table 3. As a result of non-unimodality, group comparisons were further carried out considering slow and fast spindle percentages.

**Table 3:** Median spindle frequency distribution across AHI groups, scalp locations and night sections


		**Non-OSA (7)**	**Mild OSA (11)**	**Mod OSA (10)**	**KW H**	**df**
Location	Night Period					
Frontal	total^∗∗∗^	12.6 (1.6)	12.4 (1.5)	12.1 (1.3)^*c*,*f*^	51.704	2
	I^∗^	12.4 (1.6)	12.3 (1.6)	12.1 (1.3)^*a*,*d*^	8.135	2
	II^∗∗^	12.3 (1.5)	12.3 (1.4)	12.1 (1.3)^*b*^	12.339	2
	III^∗∗∗^	12.8 (1.4)	12.5 (1.6)^*c*^	12.0 (1.2)^*c*,*f*^	48.252	2
Central	total^∗∗∗^	13.5 (1.4)	13.1 (1.7)^*c*^	12.5 (2.1)^*c*,*f*^	180.116	2
	I^∗∗∗^	13.3 (1.9)	13.0 (2.2)	12.5 (1.9)^*c*,*f*^	51.716	2
	II^∗∗∗^	13.6 (1.2)	13.3 (1.8)^*a*^	12.7 (2.1)^*c*,*f*^	46.849	2
	III^∗∗∗^	13.5 (1.3)	13.1 (1.4)^*c*^	12.3 (2.3)^*c*,*f*^	85.931	2
Parietal	total^∗∗∗^	13.7 (0.9)	13.6 (1.6)	12.9 (2.3)^*c*,*f*^	194.36	2
	I^∗∗∗^	13.6 (1.0)	13.7 (2.0)	12.6 (2.1)^*c*,*f*^	66.639	2
	II^∗∗∗^	13.7 (0.9)	13.8 (1.5)	13.3 (1.8)^*c*,*f*^	82.901	2
	III^∗∗∗^	13.8 (0.9)	13.4 (1.4)^*c*^	12.5 (2.4)^*c*,*f*^	73.002	2

AHI, Apnea-Hypopnea Index; Mod, moderate OSA; Location, scalp EEG topography; total, (I+II+III) sample; I, II and III, initial, middle and final night sections, respectively. Data presented as median (interquartile range). KW H, Kruskal-Wallis statistic; df, degrees of freedom. P-value significance as follows: ^∗^<0.05, ^∗∗^<0.01, ^∗∗∗^<0.001. Dunn’s post-hoc multiple comparisons tests as follows: *a*<0.05, *b*<0.01, *c*<0.001 for contiguous comparisons; *d*<0.05, *e*<0.01, *f*<0.001 for non-contiguous comparisons. Compared to non-OSA subjects, moderate OSA patients had significantly lower median spindle frequency in all locations and night sections under study. It should be noticed that frequency distributions were, in the large majority, non-unimodal, indicating that variations in frequency medians were only partially informative, and resulted from variability in the proportions of at least two spindle populations.

### Spindle frequency in non-OSA subjects

**Figure 3:**
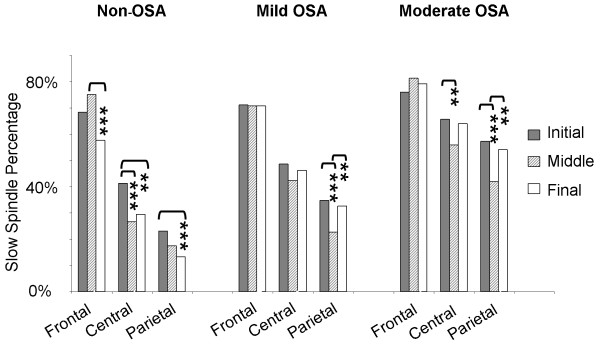
**An anterior-posterior slow spindle gradient was present, but attenuated in OSA patients, in comparison to non-OSA subjects.** For Moderate OSA, slow spindle percentage was reduced in centro-parietal, but not in frontal regions as the night progressed. In contrast, non-OSA subjects showed reduced slow spindle percentages in frontal as well as centro-parietal regions across the night.

Figure 3 shows within-group SSP according to topography in night sections I, II and III. Non-OSA subjects showed the expected frontal predominance of slow spindles, with fast spindles more prominent in parietal regions, and overlapping distributions in central scalp positions (Pearson Chi-Square =532.627;*df*2;*p*<0.001). Non-OSA subjects also displayed the expected (physiological) spindle frequency increase towards the end of the night (see also Figure 2), with a reduction in SSP and increase in fast spindle percentage in all locations under study. This was especially apparent in more anterior regions, as opposed to parietal regions, where SSP was already minor in the beginning of the night.

### Spindle frequency in mild and moderate OSA

Similarly to non-OSA subjects, mild and moderate OSA patients showed an anterior-posterior slow spindle gradient (Figure 3), with slow spindles being more prevalent in more anterior scalp locations (Pearson Chi-Squares =461.754 and =190.351, respectively; *df*2; p values <0.001). However, in comparison to non-OSA subjects, this anterior-posterior slow spindle gradient was attenuated in moderate OSA patients, due to a larger SSP in central and parietal regions, in all night sections under study. Across-group SSP comparisons according to topography and time-of-night are shown in Table 4.

**Table 4:** Slow spindle percentage in OSA according to topography and night sections


		**Total**	**Non-OSA**	**Mild OSA**	**Mod OSA**	**Chi-square**	**df**
		**(28)**	**(7)**	**(11)**	**(10)**		
Location	Night Section						
Frontal	total^∗∗∗^	72.3	66.4	71.0	78.8^*c*,*f*^	39,623	2
	I	72.1	68.3	71.2	76.1	6,041	2
	II^∗∗^	75.6	75.2	70.8	81.3^*c*^	12,242	2
	III^∗∗∗^	69.0	57.7	70.9^*c*^	79.2^*a*,*f*^	33,613	2
Central	total^∗∗∗^	47.7	32.6	45.7^*c*^	61.7^*c*,*f*^	192,429	2
	I^∗∗∗^	53.1	41.4	48.6	65.7^*c*,*f*^	50,435	2
	II^∗∗∗^	43.8	26.7	42.3^*c*^	55.9^*c*,*f*^	63,836	2
	III^∗∗∗^	46.4	29.5	46.3^*c*^	64.1^*c*,*f*^	86,224	2
Parietal	total^∗∗∗^	32.6	17.9	29.9^*c*^	50.9^*c*,*f*^	297,793	2
	I^∗∗∗^	38.9	23.2	34.7^*c*^	57.4^*c*,*f*^	101,732	2
	II^∗∗∗^	27.0	17.4	22.8	41.9^*c*,*f*^	71,609	2
	III^∗∗∗^	32.4	13.3	32.7^*c*^	54.1^*c*,*f*^	133,466	2

Mod, moderate; df, degrees of freedom; Location, scalp EEG topography; total, (I+II+III) sample; I, II and III, initial, middle and final night sections, respectively. P-value significance as follows: ^∗^<0.05, ^∗∗^<0.01, ^∗∗∗^<0.001. Significance of post-hoc inter-group comparisons (with Bonferroni correction for multiple comparisons) as follows: *a*<0.05, *b*<0.01, *c*<0.001 for contiguous comparisons; *d*<0.05, *e*<0.01, *f*<0.001 for non-contiguous comparisons.

In frontal regions, SSP was statistically similar among groups in night section I, but larger for moderate OSA in night sections II and III. Mild OSA patients had spindle frequency distributions that tended to be, in general, intermediate between non-OSA and moderate OSA patients.

### Topography-specific spindle frequency changes across the night

As the night progressed, moderate OSA patients showed spindle frequency changes that were topography-specific. In central and parietal regions, noteworthy changes to the spindle frequency curve (Figure 2) became apparent, with a relative reduction in SSP (Figure 3), especially in night section II. These changes in frequency distribution were already apparent for individual subjects (results not shown). In contrast to more posterior regions, and also in contrast to what was seen in non-OSA subjects, frontal spindles remained slow along the night in moderate OSA patients (Figures 2 and 3).

### Predictive value of slow spindle percentage in OSA

In the logistic regression analysis, frontal region, at the end of the night, was the only one to account for the outcome better than chance alone (p=0.011). The proportion of total outcome variability accounted for by the model was 43.2% . The model overall accuracy to predict OSA (with a probability of 0.5 or greater) was good (74.1% ). In night section III, for every frontal SSP unit increase, the likelihood of OSA increased by 12.1% (OR 1.121, 95% CI 1.013 - 1.239, p=0.027). BMI was not significantly associated with the outcome (OR 1.123, 95% CI 0.773 - 1.63, p=0.542). ROC analysis showed that in night section III, frontal SSP had good accuracy to differentiate between subjects with and without OSA (AUC 0.865, 95% CI 0.679 - 0.964, p<0.0001), with an SSP cut-off point of 61.9% showing 81% sensitivity and 100% specificity for OSA diagnosis within the sample.

## Discussion

This study investigated spindle frequency distribution in patients with OSA, considering scalp topography and frequency variation across the night. As the night progressed, OSA subjects persisted displaying a significant proportion of slow spindles in frontal, central and parietal regions, which was in contrast to non-OSA subjects. Concomitantly, there was a relative increase in the proportion of fast spindles in central and parietal regions, in a pattern that was similar to what was displayed by controls in frontal regions, so that only slow spindle percentage in the frontal region, in the end of the night, predicted OSA in this sample. As surface spindle frequency distribution was non-unimodal, which is in contrast to what has been reported for deep intracortical EEG sites [39], single frequency medians would not have reliably informed about these changes in proportions of two (fast and slow) spindle populations.

We interpreted these results as indicating diffuse thalamo-cortical dysfunction during sleep in OSA. They also represent evidence that dysfunction may be predominantly frontal in this context, as more posterior regions maintained, at least in part, some physiological frequency modulation throughout the night.

These findings consistent with diffuse brain dysfunction with frontal predominance are in line with results from studies relying on cognitive function assessment and/or functional neuroimaging in OSA. Several different cognitive modalities have been found to be impaired in OSA, suggesting a wide range of dysfunction [40]. These include verbal and visual learning and memory tasks, verbal fluency, attention, short-term memory, planning, programming and categorizing [41]. Treatment of 10 severe OSA patients with nasal continuous positive airway pressure (nCPAP) during 4 to 6 months normalized the majority of previously identified cognitive deficits; however, short-term memory impairment persisted, suggesting residual frontal lobe dysfunction [42]. On functional magnetic resonance imaging (MRI) of sixteen OSA patients before and after nCPAP, partial recovery of posterior parietal activation was found in contrast with a lack of prefrontal activation, and with persistent performance deficits in a verbal working memory test, suggesting a disproportionate functional impairment in dorsolateral prefrontal cortex [43]. Predominantly frontal white matter impairment has also been described in severe OSA, in a study relying on proton magnetic resonance spectroscopy [44]. In MRI studies of OSA patients, gray matter losses have been detected in different brain regions such as left hippocampus [45], frontal and parietal cortex, temporal lobe, anterior cingulate, hippocampus and cerebellum [46], although no changes have been found in one study [47]. In another MRI study, no differences were found in brain gray matter volume, but differences between OSA patients and controls were found in brain gray matter concentration in a wide range of sites, including bilateral superior frontal, frontomarginal and anterior cingulate gyri, bilateral caudate nuclei, bilateral thalami, bilateral amygdalo-hippocampi, bilateral inferior temporal gyri, and bilateral quadrangular and biventer lobules in the cerebellum [48]. Possibly, structural alterations on high-resolution magnetic resonance imaging in OSA are indications of more advanced or even irreversible neural changes [49], while functional studies relying on electrophysiology, functional neuroimaging and/or cognitive function assessment might be more sensitive to detect potentially reversible dysfunction, besides having the ability to detect permanent changes in network functionality. In this context, an electrophysiological technique such as scalp spindle frequency analysis has several advantages, including its relative simplicity, non-invasiveness, objectivity and time × cost effectiveness. It is interesting to notice that sleep spindles have been critically implicated in the mediation of NREM sleep-related memory consolidation [50-54], suggesting the possibility of a complex relationship between OSA-related brain dysfunction, spindle abnormalities and memory impairment, to be explored in future studies.

To the best of our knowledge, one previous study has directly compared spindle frequency in OSA and non-OSA subjects [8]. Subjects on that study (12 on each group) were not taking any medication. The clinical group had median AHI in the moderate range, but included mild and severe cases as well. Age span was similar to that from our subjects. In that study, spindles were selected visually and then submitted to spectral analysis. Visual selection was blindly carried out by two independent scorers working with separate hemispheres, and only synchronous, concordant spindles were included in the analysis. Inter-rater agreement was 80% (partly reflecting degree of inter-hemispheric spindle asynchrony). Spindles in proximity with obstructive respiratory events have apparently been scored, but care was taken not to mistake alpha activity for spindles. No topographic comparison was possible, as the highest sigma peak from the single EEG position (either fronto-polar, central and/or occipital) showing the highest power amplitude was analyzed for each spindle, and information from all scalp regions was pooled together. As median spindle frequency was analyzed, spindle frequency was further treated as a unimodal variable, so no information about a possible second sigma peak was available. Parietal regions were not studied. Information from the entire night was divided into initial, middle and final portions for each sleep cycle, providing a detailed map of spindle frequency changes over five NREM sleep cycles. Compared to control subjects, OSA patients were found to have lower median spindle frequency and to maintain lower frequencies throughout the night. Control subjects showed increased frequencies in the middle portion of each NREM sleep cycle towards the end of the night. To the extent to which both studies may be compared, and considering the various methodological differences, those results have been confirmed and extended by the main findings of the present study. It is noteworthy that our study relied solely on automated spindle detection, yet groups differences were consistent with those results obtained from a detailed visual analysis. Other clinical studies employing automatic methods may further help validate this approach that departs from the human visual ’gold standard’, so long as group differences are informative.

In healthy subjects, slow spindles are known to prevail in frontal regions, and to be relatively absent from parietal sites [13,14,17,55]. It was not within the scope of the present study to identify the nature of the detected parietal spindle slowing. Spindle slowing could result from general signal slowing during NREM sleep in OSA, a hypothesis not tested here. In severe OSA, general EEG slowing has been found in frontal, central and parietal regions during wake state as well as REM sleep, expressed by increases in the proportion of slow vs. fast EEG activity (delta-theta/alfa-beta ratio) [56], or confined to temporal and occipital regions during the wake state, as expressed by increases in the mean relative theta and delta power [57]. Within NREM sleep, a pattern of slower delta activity decay along the night has been verified in mild sleep disordered breathing in comparison to normal controls [12]. Spindle frequency variation within NREM cycles and along the night has been linked to sleep depth (expressed by delta activity) in healthy subjects and, at least in part, in OSA patients [8,11]. Sleep delta, or slow wave activity (SWA), is usually predominant in frontal regions. Recently, it has been proposed that the dynamics of the homeostatic sleep process, for which SWA is considered to be a phenotypical expression, is regionally specific, with faster SWA decline in parieto-occipital, and slower SWA decline in fronto-central regions [58]. The pattern of topography-specific, time-related changes in spindle frequency observed in the present study might be directly reflecting pathological changes to the sleep homeostatic process in moderate OSA. For moderate OSA patients, two different (fast and slow) spindle populations appeared to co-exist in central and parietal regions in intermediate and final night sections, suggesting an interplay of different modulatory mechanisms. Another possibility is that spindle slowing reflects frequency-specific changes in signal spectral properties directly related to thalamo-cortical circuitry dysfunction in the context of OSA, sleep fragmentation and intermittent hypoxia. As transversal studies may lack specificity to differentiate between EEG slowing due to sleepiness/homeostatic sleep pressure and more pervasive brain dysfunction, longitudinal studies (e.g. before and after positive airway pressure treatment) might clarify this issue.

Whether some degree of fast alpha intrusion could be responsible for the increased finding of phasic components in the slow sigma/fast alpha frequency range also deserves consideration. Classical arousals, which need to last at least 3s, were systematically excluded and are unlikely to be influencing our results. However, alpha activity with several different temporo-spatial patterns has been shown to be an integral part of NREM sleep in physiological as well as pathological conditions [59]. Alpha rhythms are traditionally believed to indicate wakefulness [60]. The electrophysiological origin of different sleep alpha patterns is still unaccounted for. In a recent work [61], drivers with severe fatigue during wakefulness expressed high numbers of short-time (less than 1s) EEG alpha bursts believed to represent fragments of waking alpha activity, and typically occurring in drowsiness and early wake-sleep transition. These alpha bursts (which the authors called ’alpha spindles’) were predominantly expressed over occipital regions, but they were also present, to a lesser extent, over parietal, central and frontal sites. Interestingly, their frequency was slower in more anterior regions, and faster in more posterior locations. In the chronically implanted cat, Steriade and McCarley [62] describe the transition between wake and sleep as the short period when surface sleep spindles appear intermingled within the steady-state of waking, before increasing in amplitude and occurring in association with slow wave activity, as sleep intervenes. Either by visual analysis or through a signal decomposition approach like MP, short alpha bursts would be similar to sleep spindles. In a setting where sleep maintenance processes responsible for sleep spindle production have to compete with arousal mechanisms, believed to be implicated in alpha activity generation, the distinction between these two types of activity may be compromised.

The rich and complex subject of topographical spindle frequency dynamics has been little studied in the specific context of brain pathology. The present study only provides a limited view into such dynamics. Spindles originate in the thalamic reticular nucleus, which induces discharges in thalamo-cortical circuits, ultimately transferred to cortical neurons. While spindles may be identified in decorticated animals [63], neocortex plays a fundamental role in spindle propagation and modulation [62]. Traditionally, studies in cats and rodents considered only one spindle type, and studies in humans considered the existence of two spindle types, with slow spindles prevailing on more anterior brain regions, and fast spindles prevailing on parietal locations. These concepts have been challenged lately. At least two spindle types have now been identified in rats [64]. Internal (within-spindle) frequency variation has been demonstrated in rats [64] as well as in humans [65], and systematically measured in humans [66,67]. It has been shown that single spindles tend to decelerate over time [67]. In humans suffering from epilepsy, a depth intracortical EEG study has shown widespread spindling activity over several different areas, with smooth spindle frequency and density changes along the caudo-rostral axis, from fast frequent posterior to slower and less frequent anterior spindles [39]. A magnetoencephalography (MEG)-EEG study has shown a temporospatial frequency evolution from posterior-fast to anterior-slow generators commonly occurring during single spindles [68]. Another MEG-EEG study of visually-detected spindles identified a mixture of activities related to slow and fast spindles over pre-central as well as post-central areas, suggesting a unifying network underlying spindles over central areas, and that slow and fast spindle activity may represent a single event in global thalamo-cortical coherence [69]. Differences in temporal activation between hemispheres have been linked to fast spindle interhemispheric amplitude asymmetries in another MEG-EEG study [70]. Relying on automated spindle detection over multiple brain regions in a depth intracortical study of neurosurgical patients, Tononi and cols. have demonstrated that local, as opposed to globally occurring spindles, constitute the majority of events in natural human sleep [65]. Clearly, the traditional concept of slow frontal and fast parietal spindles is an oversimplification of a much finer process, which is only beginning to be unveiled.

A number of limitations need to be considered in this study. Control subjects were not healthy subjects, they were snorers with other sleep complaints who might suffer from upper airway resistance syndrome. However, as respiratory effort-related arousals have been shown to negatively impact sleep microstructure [71], this fact would tend to reduce differences between OSA patients and controls. Age could be another factor potentially diminishing inter-group differences. There was a non-significant trend towards older age in Mild and Moderate OSA groups, where spindle frequency was lower, whereas spindle frequency is expected to increase with age. More importantly, subjects were not free from medication, in spite of the exclusion of benzodiazepine use, well known to affect sleep spindles [72]. Recently, a tricyclic agent (desipramine) was shown to reduce spindle sleep time in rats [73]. Reboxetine, a selective noradrenaline reuptake inhibitor, has been shown to increase number and density of fast spindles (>13*Hz*) in humans [74]. Five subjects in this study were taking tricyclic agents. They were evenly distributed among groups (1 non-OSA, 2 mild and 2 moderate OSA). However, the extent to which this medication use may have influenced our results is not fully known. Ours was an exploratory study in a realistic clinical setting, where several potential confounding variables could not be controlled. Results should be interpreted accordingly, and confirmation by other studies may be warranted. Another limitation was the exclusion of severe OSA patients due to excessive noise and sleep fragmentation surrounding apneic events. A different study design, for instance focusing on the occurrence of spindles during the apneic event, might be better suited to address that population. The main findings from the present study are expected to be confirmed in severe OSA.

## Conclusion

In conclusion, OSA patients showed significant, topography-specific changes in sleep spindle frequency across the night, in a pattern consistent with diffuse, predominantly frontal thalamo-cortical dysfunction. It is reasonable to speculate that spindle changes may be implicated in OSA-related memory dysfunction, either causally or as an epiphenomenum of abnormal underlying neural processes. Spindle frequency abnormalities are not specific to a disease type, and they are not proposed here as a diagnostic tool. Their predictive value illustrates their sensitive power, which indicates this variable to be a useful electrophysiological marker of brain dysfunction in OSA. We also believe that the computational workflow implemented in this study could be easily extended to investigate other conditions in an automated manner, using different grid or cloud infrastructures available to scientists at low costs.

## Authors’ contributions

SVS, DZC, GJLG and ELSH carried out the experiments. SVS and GJLG wrote the first draft of the manuscript. NL, ELSH, GJLG, SVS, and DZC participated in the study design, performed the statistical analysis, and helped improve the manuscript draft. NL is also responsible for the massive computing analysis. All authors analyzed the experiments, read and approved the final manuscript.

## Supplementary Material

Additional file 1 **Supplementary figure showing the procedure employed for MP amplitude threshold selection.** Matching Pursuit performance was tested on another sample (training dataset) pertaining to 9 healthy young subjects, where 513 sleep spindles had been visually identified during NREM sleep stage 2 (same database used in Schonwald et al; Benchmarking Matching Pursuit to find sleep spindles. Journal of Neuroscience Methods 2006, 156:314-321). The test was carried out on the C3-A2 channel with MP parameters used in this study (number of atoms in the dictionary, frequency and duration limits) and Receiver-Operator Characteristics (ROC) curves were built according to voltage threshold. Additional curves show correspondence between specificity, accuracy and higher amplitude atom percentage (top atoms) according to total atoms detected. An MP 20*%* amplitude threshold corresponded to 96*%*specificity on the training dataset. Click here for file
