# Supplementary figures and images for: Topography-specific spindle frequency changes in Obstructive Sleep Apnea
Source: BMC Neurosci. 2012 Jul 31;13:89. doi: 10.1186/1471-2202-13-89 (PMC3496607; doi:10.1186/1471-2202-13-89)

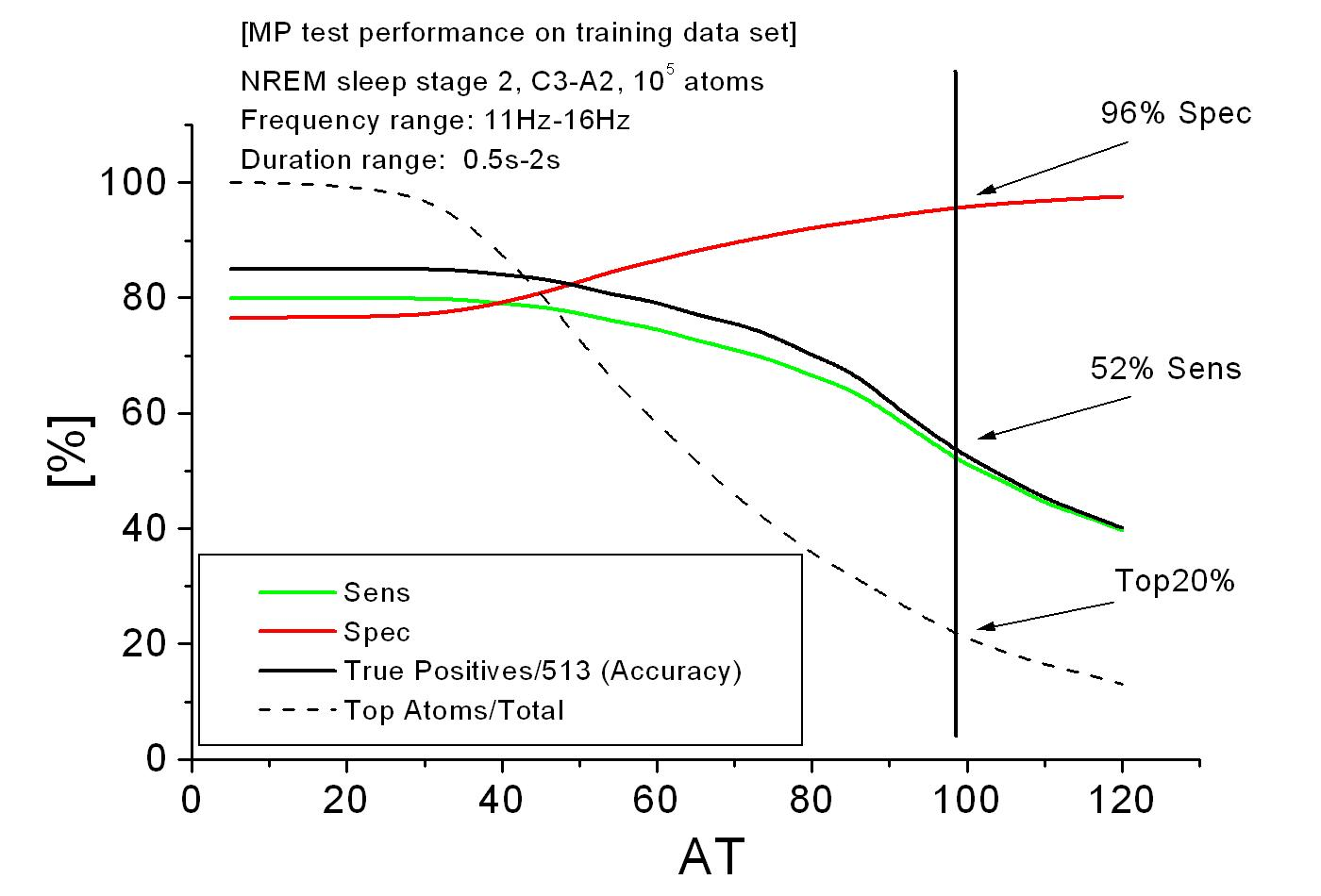

Supplement: Additional file 1 — Supplementary figure showing the procedure employed for MP amplitude threshold selection. Matching Pursuit performance was tested on another sample (training dataset) pertaining to 9 healthy young subjects, where 513 sleep spindles had been visually identified during NREM sleep stage 2 (same database used in Schonwald et al; Benchmarking Matching Pursuit to find sleep spindles. Journal of Neuroscience Methods 2006, 156:314-321). The test was carried out on the C3-A2 channel with MP parameters used in this study (number of atoms in the dictionary, frequency and duration limits) and Receiver-Operator Characteristics (ROC) curves were built according to voltage threshold. Additional curves show correspondence between specificity, accuracy and higher amplitude atom percentage (top atoms) according to total atoms detected. An MP 20% amplitude threshold corresponded to 96%specificity on the training dataset. [file 1471-2202-13-89-S1.tiff]
